# Supplementary material for: How risk-prone are people when facing a sure loss? Negative interest rates as a convenient conceptual framework
Source: Psychon Bull Rev. 2021 May 4;28(5):1715–25. doi: 10.3758/s13423-021-01921-0 (PMC8095218; doi:10.3758/s13423-021-01921-0)
Supplement: Supplementary file 1 — (DOCX 4286 kb) [file 13423_2021_1921_MOESM1_ESM.docx]

**How Risk-Prone are People when Facing a Sure Loss?**

**Negative Interest Rates as a Convenient Conceptual Framework**

Emir Efendić^12^*, Olivier Corneille^1^, Catherine D’Hondt^1^ & Rudy De Winne^1^

^1^University of Louvain

^2^Maastricht University

Author note: This work is supported by the joint research grant ARC; Grant n.: ARC 18/23.089 from the FWB awarded to OC and CD.

Author contributions: EE and OC developed the study concept and study design. Data collection and analyses were planned and performed by EE after discussions with all authors. All authors wrote the manuscript.

*Correspondence concerning this article should be addressed to Emir Efendić, Maastricht University, School of Business and Economics, MSCM, Tongersestraat 53, 6211LM Maastricht, The Netherlands. E-mail: [efenemir@gmail.com](mailto:efenemir@gmail.com)

The authors declare no competing interests.

**Abstract (185 words)**

People occasionally face sure loss prospects. Do they seek risk in search of better outcomes or contend with the sure loss and focus on what is left to be saved? We addressed this question in three experiments akin to a negative interest rate framework. Specifically, we asked participants to allocate money (Experiments 1 and 2) or choose (Experiment 3) between two options: i) a loss option where, for sure, they would end up with less, or ii) a mixed gamble with a positive expected outcome, but also the possibility of an even larger loss. Risk aversion (i.e., choosing the sure loss) ranged from 80% to 36% across the three experiments, dependent on varied sizes of sure losses or expected outcomes. However, overall, the majority (> 50%) of allocations and choices were for the sure loss. Our findings indicate a tolerance for sure losses at the expense of mixed gambles yielding much better expected outcomes. We discuss the implications of this sure-loss tolerance for psychological research, its implications in terms of (cumulative) prospect theory, and what the results mean for the implementation of negative interest rates.

*Keywords:* Risk-Taking; Sure Loss; Certainty effect; Negative Interest Rates

**How Risk-Prone are People when Facing a Sure Loss?**

**Negative Interest Rates as a Convenient Conceptual Framework**

Psychological research has largely investigated how people react to bright future prospects, where they typically have to choose between a rather comfortable option (e.g., a Marshmallow or $100 tomorrow) and an even brighter future (e.g., two Marshmallows or $110 in a week; Bartels & Rips, 2010; Mischel, 2014). In that research, people usually ask a premium for bearing with the psychological cost of their patience. Yet, people occasionally face sure loss prospects. For instance, negative interest rates (NIR) entail a future sure loss if money is left on a bank account. Surprisingly, little empirical research exists on how people deal with such decisions. Would they seek risk or accept to actually *pay* for their patience?

**Deciding between a sure loss and a gamble.**

To address our question, we contrast the prospect of a sure loss to the uncertainty of a mixed gamble (i.e., a risky option with a high positive expected return that offers a possibility of a large gain but also of a loss, larger than the sure one). Previous research has presented people with related choice contexts, however, to the best of our knowledge, the choice context we use here is unique in the literature.

One of the dominant theories explaining decision-making under risk and uncertainty is (cumulative) prospect theory (Kahneman & Tversky, 1979; Tversky & Kahneman, 1992). The first key element of CPT is the shape of the value function (concave for gains, convex for losses). Crucially, the value function is steeper for losses predicting loss aversion. The second key element is the probability weighting function as people are predicted to overweight small, but underweight moderate and high probabilities.

In our decision context, akin to current NIR implemented on saving accounts, the sure option entails a *necessary* loss. In previous research, the sure option entailed a zero outcome. Specifically, it has been shown that, when faced with a choice between a sure option that entails a $0 outcome and an attractive yet risky mixed gamble (e.g., 50% chance to gain $2,000 or 50% chance to lose $500), most people prefer the sure $0 (Redelmeier & Tversky, 1992; Tversky & Kahneman, 1992). These findings point to *risk aversion*, i.e., a tendency not to choose the mixed gamble. The psychological mechanism considered responsible for this is loss aversion, whereby losses loom larger than gains (Kahneman & Tversky, 1979). Thus, when offered a choice of a mixed gamble with the alternative being zero, people are predicted to be risk averse.

Unlike the contexts described above, we propose to present people with a sure option that entails a sure loss. Previous studies had participants face sure losses. One such case is when the prospects were only in the loss domain (i.e., both the sure and the gamble option entailed losses). For example, when people were asked whether they would prefer having a certain loss of $10 or having a 50% chance of losing $5 vs. a 50% chance of losing $15, most preferred the latter option, i.e., they were risk seeking (Leclerc et al., 1995). The shape of the value and probability functions in prospect theory predict risk seeking for losses of moderate probability.

In cases with more extreme (i.e., close to 0% and 100%) probabilities, the weighting function allows for different predictions. The underweighting of high probabilities contributes to risk aversion in choices between probable and sure gains. However, this contributes to *risk seeking* in choices between probable and sure *losses*. Thus, when faced with a prospect of a sure loss, people tend to take risks meaning that they prefer a larger uncertain loss to a smaller, but certain loss (Tversky & Kahneman, 1992). Such a pattern of preferences has been deemed the *certainty effect* (Kahneman & Tversky, 1979), predicting a strong aversion to sure losses (Ruggeri et al., 2020).

Some previous research has contrasted a sure loss to gambles where one option entails a loss, while the other entails a zero outcome (e.g., 20% chance to lose 20 vs. 80% chance of no loss). Because these gambles entail a zero option they may not be considered mixed (Abdellaoui et al., 2007). A gamble is commonly said to be mixed if there is a possibility of a loss *and* a possibility of a gain (Baltussen et al., 2006; Ert & Erev, 2008; Wu & Markle, 2008). Of critical importance however, beyond terminological usage, in our current experiments we purposely avoided zero options because we wanted to make sure that the risky mixed gamble had a high (and crucially positive) expected return, but also the possibility of a loss higher than the sure one. Some research did contrast a sure loss to a mixed gamble (with non-zero options for gains and losses), however the gamble did not have a positive expected return (Erev et al., 2010).

To sum up, our choice context is unique in that: i) the sure option is a loss, ii) the non-zero options mixed gamble has a possibility of a loss higher than the sure one, and iii) the mixed gamble has a positive expected return. In doing so, our decision setting allows testing two conflicting predictions. On the one hand, people’s aversion to the possibility of an even larger loss if choosing the gamble ought to lead to risk aversion and so to acceptance of the sure loss. On the other hand, the presence of a sure loss ought to lead to risk seeking behavior (and a preference for the gamble).

As it appears, beyond its theoretical implications, the decision context we examine here is relevant to situations of large economic downturn and questions of safety. For instance, with NIR, a person could either have the option of leaving some money, say $100, on a bank account where in a year they will lose $5 for sure and have $95. Alternatively, the money could be invested in a stock where in a year they could have, for instance, 40% chance to lose $10 but a 60% to gain $20.

**Experiment 1**

**Method**

In Experiment 1, participants could allocate a proportion of money between a sure loss option at -0.5% and a risky mixed gamble. We keep the sure loss option constant (i.e., one would lose 0.5% of the allocated money) while changing the expected outcome of the risky option. This implies varying risk premiums (i.e., the expected excess return provided by investing in the risky over the sure loss option). For exploratory purposes, we manipulated three additional factors: the amount of money one has at disposal, how people come about the money, and the order in which the risky options are experienced.

We explored the role of these factors as they may provide insights into boundary conditions to our current question. The size of money at a person’s disposal can impact the risk, but also the loss they are willing to take on. We know for example that loss aversion can reverse for small amounts of money (Harinck et al., 2007). Similarly, a magnitude effect is often found whereby risk aversion increases with payoff size – this has been labeled as “relative risk aversion” (Holt & Laury, 2002). We were also interested in how people came about the money. Losses on savings can be particularly devastating, while it is known that people are more risk seeking in the presence of a prior gain (i.e., the house money effect; Thaler & Johnson, 1990).

The experiment had a 2 (*Money*: Saved vs. Unexpectedly Received) x 2 (*Amount*: $1,000 vs. $40,000) x 7 (*Risky option expected outcome*: -0.5% vs. 0% vs. 0.5% vs. 1% vs. 3% vs. 5% vs. 10%) x 2 (*Order* of risky option: Upward from -0.5% to 10% vs. Downward from 10% to -0.5%) mixed design. Money and order were between-, while amount and risky option expected outcome were within-subject factors.

**Participants**

A hundred and forty-eight individuals from the US were recruited on Prolific. We aimed to recruit 30^[[1]](#footnote-1)^ participants per between-subject condition. After excluding participants who failed an attention check at the beginning of the study and those who did not complete the entire study, data from 119 participants were retained (61% female; *Med*_Age_ = 30, *IQR*_Age_ = 14).

**Procedure**

Participants started by filling in a consent form. If they completed the first attention check correctly, they were presented with the instructions (read instructions here: <https://osf.io/3zfwu/?view_only=e145857513e740f08c27299268d3b554>). Next, participants were asked about their comprehension of the instructions. Eight participants who answered incorrectly were presented with the instructions again and asked to be more attentive. Participants were informed that a new policy was introduced where people are required to pay to keep money in the bank. They were asked to decide what proportion of either saved or unexpectedly received money to allocate for the next one year between two options. The first was the sure loss option that always guaranteed a loss of 0.5% in one year. The second was the risky option. We presented the gamble using a symmetric distribution with nine possible outcomes, each associated with a probability of occurrence. The middle of the distribution was associated with the highest probability and thus represented the expected outcome of the gamble (see Table 1).

[Insert Table 1 about here]

| ***Table 1.*** Payoffs for each of the seven risky options in Experiment 1. Volatility is kept constant at 17%. The expected outcome (highlighted in gray) is associated with the highest probability of occurrence (26%). Important to note that as the expected outcome increases, losses associated with the risky option decrease while gains increase (from Risk 1 to 7). | | | | | | | |
| --- | --- | --- | --- | --- | --- | --- | --- |
|  | **Risk distribution for seven risky options** | | | | | | |
| **Probability of occurring** | **Risk 1** | **Risk 2** | **Risk 3** | **Risk 4** | **Risk 5** | **Risk 6** | **Risk 7** |
| 2% | -40.5% | -40% | -39.5% | -39% | -37% | -35% | -30% |
| 5% | -30.5% | -30% | -29.5% | -29% | -27% | -25% | -20% |
| 12% | -20.5% | -20% | -19.5% | -19% | -17% | -15% | -10% |
| 18% | -10.5% | -10% | -9.5% | -9% | -7% | -5% | 0% |
| 26% | -0.5% | 0% | +0.5% | +1% | +3% | +5% | +10% |
| 18% | +9.5% | +10% | +10.5% | +11% | +13% | +15% | +20% |
| 12% | +19.5% | +20% | +20.5% | +21% | +23% | +25% | +30% |
| 5% | +29.5% | +30% | +30.5% | +31% | +33% | +35% | +40% |
| 2% | +39.5% | +40% | +40.5% | +41% | +43% | +45% | +50% |

Participants were tasked with allocating money into these two options for seven risk distributions for two different amounts of money, totaling 14 allocation decisions. Allocations for the two starting amounts (i.e., $1,000 and $40,000) were made in random blocks. An example of the allocation decision question is presented in Figure 1. To illustrate the payoffs in the experiment, participants were presented with a chart. The chart displayed the probabilities of each possible payoff occurring if 100% of the amount at disposal was allocated to the gamble (to view all of the charts used in the experiment, see the *stimuli* section on the OSF page). The expected outcome was centered on the middle bar and associated with the highest probability of occurring.

[Insert Figure 1 about here]


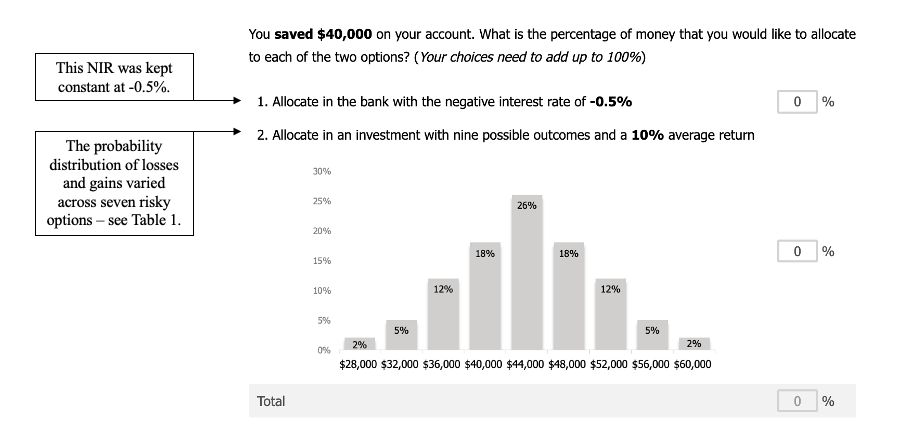


***Figure 1*. Illustration of an allocation decision setup used in Experiment 1.** The picture shows an example where the amount was $40,000 and the money was saved. The risky option with a 10% expected outcome (i.e., Risk 7 in Table 1) is displayed here. In each trial, the sure loss option (i.e., option “1”) was kept constant at -0.5%, while the chart representing the distribution of the losses and gains associated with the mixed-gamble option (i.e., option “2”) changed.

The volatility (i.e., the spread of the risk) was kept constant across all risky options at 17%. It should be noted here that as the expected outcome associated with the mixed-gamble increases, lower losses are possible (see Table 1). That is, in Risk 7 (expected outcome 10%), losses are smaller, than in Risk 1 (expected outcome -0.5%). Furthermore, overall probabilities of a loss occurring were highest in Risk 1 (63%), were the same in Risk 2 to 6 (37%), and lowest in Risk 7 (19%). Our dependent variable was how much money (in percentages) was allocated into the sure loss option. Because status quo bias can lead to higher loss aversion (Gal, 2006), we made sure not to frame either of the two options as the default (also done in Experiments 2 and 3) and we kept the time of the potential outcome constant, i.e., in one year one would end up with a sure loss or in one year one would end up with either a gain/loss from the mixed gamble (also done in Experiments 2 and 3). At the end, participants responded to several demographic variables (i.e., education, income, financial experience, saving – a percentage estimate of how much they saved over the last three years, and gender).

**Results**

We regressed the percentages allocated to the sure loss option on the four factors and their interactions. This was a multilevel model where the interaction between the within-subject factors (amount and expected outcome) was allowed to vary for each participant (treated as a random factor). The results show that there was a main effect of risky option expected outcome, *b* = -5.38, *SE* = 0.49, 95% CI [-6.34, -4.42], *t*(114.99) = -10.98, *p* < .001, *d*z = 1.0^[[2]](#footnote-2)^. Participants allocated less money into the sure loss option as the expected outcome of the risky option (and the risk premium) increased. There was also a main effect of order, *b* = -19.37, *SE* = 5.85, 95% CI [-30.84, -7.90], *t*(114.99) = -3.31, *p* = .001, *d*z = .30. Participants allocated more money (*M =* 54.6%) into the sure loss option in the downward than in the upward (*M =* 47.1%) order. These two effects were qualified by an interaction, *b* = 3.20, *SE* = 0.98, 95% CI [1.28, 5.12], *t*(114.99) = 3.926, *p* = .001, *d*z = .30 (see Figure 2). No other effects were significant. We decomposed the interaction by the order factor. Looking at the upward order first, there was a significant effect of risky option expected outcome, *b* = -3.71, *SE* = 0.59, 95% CI [-4.86, -2.55], *t*(59) = -6.301, *p* < .001, *d*z = .58 with the amount of allocations to the sure loss option decreasing with larger expected outcomes. Looking at the downward order, we found the same effect, although much stronger, *b* = -7.03, *SE* = 0.77, 95% CI [-8.54, -5.52], *t*(58) = -9.15, *p* < .001, *d*z = .84.

[Insert Figure 2. about here]

**
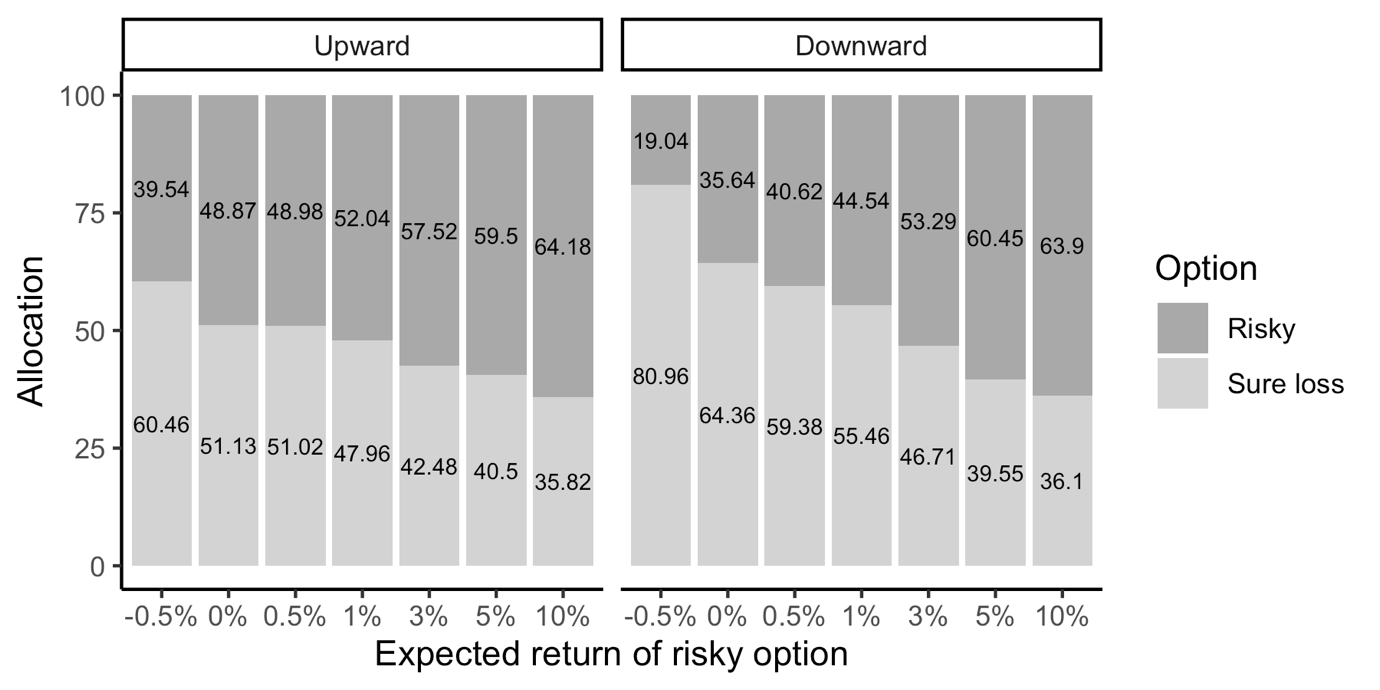
**

***Figure 2****.* **Mean percentage of money allocated to the sure loss and risky options in Experiment 1 as a function of expected outcome of the risky option and the order of the expected outcome presentation.** Numbers inside the bar charts represent the raw percentage of money allocated.

The results do not change overall when adding the demographic variables into the model. However, after including these covariates there is now an additional effect of Money, *b* = 12.82, *SE* = 5.87, 95% CI [1.31, 24.33], *t*(108.53) = 2.18, *p* = .03, *d*z = .20. Opposite to the house money effect, when money was unexpectedly received, participants allocated more (*M* = 54.9%) into the sure loss option. There was also a negative relationship with education, *b* = -4.76, *SE* = 1.89, 95% CI [-8.46, -1.06], *t*(110.00) = -2.52, *p* = .013, *d*z = .23; more educated participants allocated less money to the sure loss option.

Unsurprisingly, we observed more risk-taking with higher expected outcomes (i.e., higher risk premiums). Critically, however, in the majority of choice options (4 out of 7), participants were indifferent or risk-averse, allocating more money to the sure loss option even when the mixed-gamble option entailed a much higher expected outcome. For risk premiums above 1% however, participants switch their preferences (i.e., they allocate more than 50%) to the risky option.

**Experiment 2**

**Method**

We implemented four changes in Experiment 2, which was otherwise similar to Experiment 1. First, we only examined the savings condition. Second, we varied and lowered the sure loss option (i.e., made it more negative). Third, we kept the overall probabilities of gains and losses of the mixed gamble constant (at 63% and 37%, respectively). The overall probabilities varied across the risky options in Experiment 1. We wanted to make sure this does not impact our investigation of sure loss tolerance as previous findings have indicated people avoid, not just high losses, but losses with a higher probability of occurring (Payne, 2005). Fourth, we kept the risk premium constant at 11%. In Experiment 1, the expected returns (i.e., risk premiums) changed, which could have impacted overall risk-taking preparedness. Keeping risk premiums constant meant that with higher sure losses, the risky option implied lower gains and higher losses (see Table 2).

[Insert Table 2 about here]

| Table 2. Payoffs for each of five risky options in Experiment 2 as a function of the sure losses. The expected returns (in gray) depended on the sure loss because the risk premium was kept constant at 11%. It should be noted (i) that as the sure loss increases, the gains associated with the risky option decrease while the losses associated with the risky option increase, (ii) the overall probability of ending up with a gain or with a loss is kept constant (at 63% and 37%, respectively). | | | | | |
| --- | --- | --- | --- | --- | --- |
| **Probability of occurring** | **Risk distribution of the five risky options as a function of the sure loss** | | | | |
|  |  | | | | |
|  | **Sure loss 1**  **0%** | **Sure loss 2**  **-0.5%** | **Sure loss 3**  **-1%** | **Sure loss 4**  **-3%** | **Sure loss 5**  **-5%** |
| 2% | -37% | -37.5% | -38% | -40% | -42% |
| 5% | -25% | -25.5% | -26% | -28% | -30% |
| 12% | -13% | -13.5% | -14% | -16% | -18% |
| 18% | -1% | -1.5% | -2% | -4% | -6% |
| 26% | +11% | +10.5% | +10% | +8% | +6% |
| 18% | +23% | +22.5% | +22% | +20% | +18% |
| 12% | +35% | +34.5% | +34% | +32% | +30% |
| 5% | +47% | +46.5% | +46% | +44% | +42% |
| 2% | +59% | +58.5% | +58% | +56% | +54% |

The experiment had a 2 (*Amount*: $1,000 vs. $40,000) x 5 (*Sure loss*: 0% vs. -0.5% vs. -1% vs. -3% vs. -5%) x 2 (*Order* of loss presentation: upward from 0% to -5% vs. downward from -5% to 0%) mixed design. Order was the only between-subject factor. Participants thus completed 10 allocation decisions. The instructions were similar to those of Experiment 1 (for full instructions, see: <https://osf.io/3zfwu/?view_only=543c8c0a03744cd1825b6b12e5b546d4>).

**Participants**

Seventy-four individuals from the US were recruited on Prolific. After excluding participants who failed an attention check at the beginning of the study and those who did not complete the entire study, data from 60 participants were retained. One participant made negative allocation decisions (e.g., allocating -5% when the sure loss was -5% and so on). Presumably, this participant did not understand the instructions and was excluded. We thus retained 59 participants (61% female; *Med*_Age_ = 31, *IQR*_Age_ = 13.5).

**Results**

The same analysis strategy as in Experiment 1 shows that there was a main effect of amount, *b* = 7.73, *SE* = 3.57, 95% CI [0.74, 14.73], *t*(57.00) = 2.17, *p* = .034, *d*z = .28. A higher proportion of money is allocated to the sure loss option for the $40,000 (52.7%) amount than for the $1,000 (45.1%) amount. There was also a main effect of sure loss, *b* = -6.28, *SE* = 0.88, 95% CI [-8.01, -4.55], *t*(57.00) = -7.13, *p* < .001, *d*z = .93. Participants allocated less money to the sure loss option as it became more negative. No other effects were significant. Most importantly, we again observed that for most (3 out of 5) choice options, people were indifferent or risk-averse, preferring to allocate more money into the sure loss (see Figure 3). In contrast to Experiment 1, we found no effect of order and we observed a difference due to starting amounts. People took less risks with larger amounts of money. Adding the demographic information into the model, the results remained the same. There was, however, a negative relationship with financial experience. Those who said they had more financial experience, allocated less into the sure loss option, *b* = -12.13, *SE* = 5.18, 95% CI [-22.29, -1.97], *t*(52.00) = -2.34, *p* = .023, *d*z = .30.

[Insert Figure 3 about here]

**
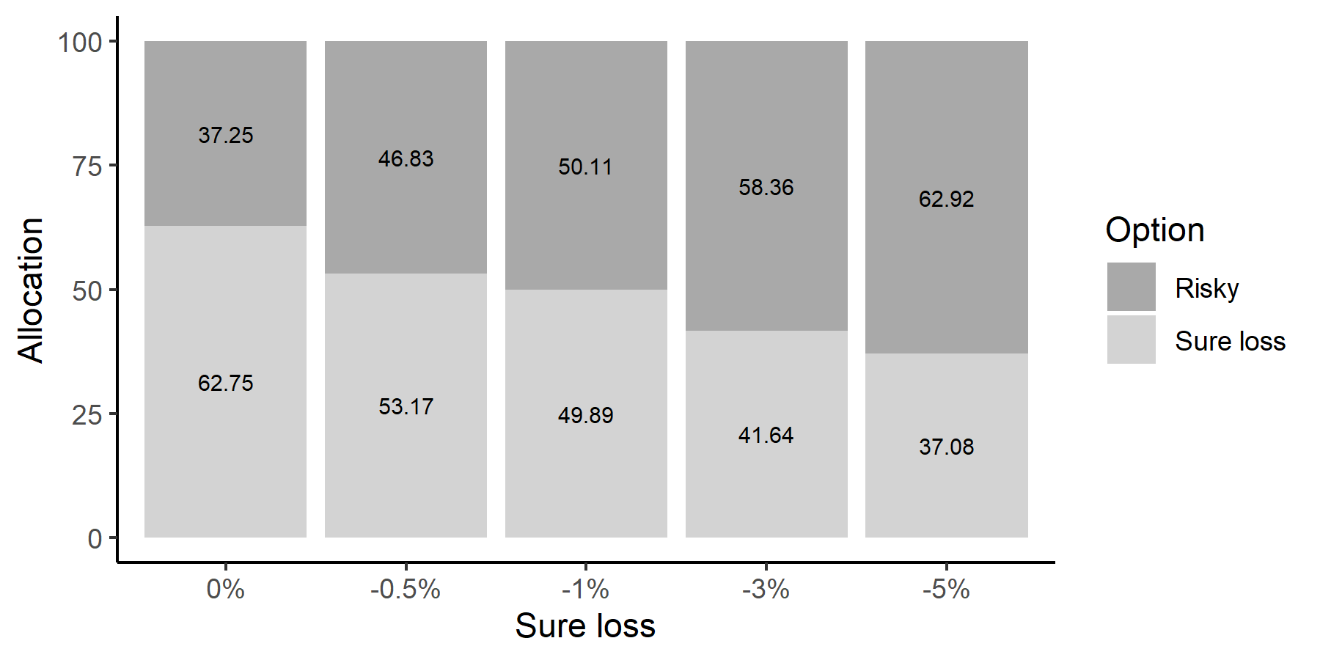
**

***Figure 3***. **Mean percentage of money allocated to the sure loss and risky options in Experiment 2 as a function of the sure loss.** Numbers inside the bar charts represent the raw percentages.

**Experiment 3**

**Method**

Experiment 3 consisted of another robustness check for the effects observed in Experiments 1 and 2. As such, instead of using allocation decisions and having the participants contend with percentage losses and gains (e.g., lose -3%), we asked them to make clear-cut choices (i.e., *either* choose a sure loss *or* a mixed gamble) and we presented losses and gains in numerical values (e.g., lose $5) instead of percentages (e.g., lose -3%). We also used smaller amount sizes (i.e., $100 and $500) as the losses used in the previous experiments could have been perceived as too large given the larger amounts. Finally, the specific context in which we put individuals in the previous experiments (i.e., a NIR environment) may have played a role perhaps cuing people that sure losses are widespread. As such, in Experiment 3, we varied the decision context, presenting three sets of instructions to different groups: a context similar to previous two experiments, a base context with no additional information, and a context reminding people of the current economic state brought about by the COVID-19 pandemic (see procedure below). We expected a robust replication for loss tolerance across these three contexts but were curious whether indirectly reminding participants of the current economic turmoil due to the Covid-19 outbreak might increase sure loss tolerance. Probabilities of gains and losses of the mixed gamble were held constant (at 60% and 40%, respectively). The risk premium was held constant at 15%. Again, similar to Experiment 2, keeping risk premiums constant meant that with higher sure losses, the risky option implied lower gains and higher losses (see Table 3). Because we also had a sure loss that equaled -15%, the expected return of this option was 0 (again, because the risk premium was held constant).

[Insert Table 3 about here]

| Table 3. Payoffs for each of six risky options as a function of the sure losses in Experiment 3. The risk premium was kept constant at 15%. It should be noted (i) that as the sure loss increases, the gains associated with the risky option decrease while the losses associated with the risky option increase, (ii) the overall probability of ending up with a gain or with a loss is kept constant (at 60% and 40%, respectively), (iii) because the risk premium is held at 15%, the -15% sure loss meant that the expected return was 0 for this option and (iv) here, the payoffs are given in percentages for convenience as there are two amounts while in the experiment they were given as numerical values. | | | | | | |
| --- | --- | --- | --- | --- | --- | --- |
| **Probability of occurring** | **Risk distribution of the six risky options as a function of the sure loss** | | | | | |
|  |  | | | | |  |
|  | **Sure loss 1**  **-0.5%** | **Sure loss**  **2**  **-1%** | **Sure loss 3**  **-3%** | **Sure loss 4**  **-5%** | **Sure loss 5**  **-10%** | **Sure loss 6**  **-15%** |
| 40% | -30.5% | -31% | -33% | -35% | -40% | -45% |
| 60% | +44.5% | +44% | +42% | +40% | +35% | +30% |

The experiment had a 2 (*Amount*: $100 vs. $500) x 6 (*Sure loss*: -0.5% vs. -1% vs. -3% vs. -5% vs. -10% vs. -15%) x 3 (*Context*: NIR vs. Base – where participants were simply provided with a choice between a sure loss and a mixed gamble vs. Covid-19). Context was the only between-subject factor. We did not look at order of the sure loss presentation as there was no effect of order in the previous experiment. However, for each participant it was randomly determined whether they were presented with the sure losses in an increasing (i.e., from -0.5% to -15%) or decreasing order (i.e., from -15% to -0.5%)^[[3]](#footnote-3)^. Participants thus made 12 choices in total. It was also explicitly highlighted to participants to treat each choice as independent from one another to avoid the slight possibility that they may infer the choices are connected and that the losses are continuous.

**Participants and procedure**

Three-hundred and thirty^[[4]](#footnote-4)^ individuals from the US were recruited on Prolific. No participants were excluded (61% female; *Med*_Age_ = 31, *IQR*_Age_ = 13.5). The overall procedure was similar to Experiment 2, the main difference being the context instructions and the choice options were now worded differently (i.e., choice instead of allocation and gains/losses were presented in numerical, rather than percentage terms). For full instructions, see: <https://osf.io/2xr58/?view_only=3d0fb8404ec14d2f8156ec66392baafa>. In the NIR context, the instructions were similar to the previous two experiments while the choice options were (The X’s changed dependent on the sure loss and the mixed gamble payoffs):

*1:* Put in the bank with the negative interest rate of -X%. Thus, in a year you will lose $X for sure and have $X.

*2:* Invest. Thus, in a year, there is a 40% chance to have $X (and so you would lose $X) or a 60% chance to have $X (and so you would gain $X).

Participants in the Base context were simply asked to make several choices pertaining to the financial decision-making. They were told they had some money and they had to choose what to do with it for the next 1 year, having to choose between two options worded as:

You have $X. Which option do you choose?
*1:* Choose this option and, in 1 year, you will lose $X for sure and have $X

*2:* Choose this option and, in 1 year, you have 40% chance to have $X (and so you would lose $X) or a 60% chance to have $X (and so you would gain $X).

In the Covid-19 context, the instructions were the same as in the Base context beside one paragraph that reminded participants to “…please reflect on the current economic climate, worsened by the devastating COVID-19 pandemic…make each of the choices below in light of the current economic situations as a background context for making your decisions” (see exact text on the OSF page).

**Results**

We used a logistic regression model that regressed the three factors and their interactions on the choice of sure loss (0) or risky gamble (1); the interaction between the within-subject factors (amount and sure loss) was allowed to vary for each participant (treated as a random factor). The context factor was sum contrast coded (C1: NIR = 1; Base = 0, Covid = -1 and C2: NIR = 0; Base = 1, Covid = -1). The results are reported in Table 4 below.

[Insert Table 4, about here]

***Table 4.*** 95% CI’s, odds ratios and the *p* values of the logistic regression model in Experiment 3, regressing the Context, Amount, Sure loss, and their interactions onto choice (i.e., either a sure loss or a risky mixed gamble).

| *Predictors* | *Odds Ratios* | *CI* | *p* |
| --- | --- | --- | --- |
| (Intercept) | 0.15 | 0.06 – 0.40 | **<0.001** |
| Context C1 | 0.21 | 0.06 – 0.82 | **0.025** |
| Context C2 | 2.34 | 0.61 – 9.02 | 0.217 |
| Amount | 4.66 | 1.39 – 15.63 | **0.013** |
| Sure loss | 1.37 | 1.16 – 1.61 | **<0.001** |
| Context C1 * Amount | 1.26 | 0.25 – 6.23 | 0.778 |
| Context C2 * Amount | 1.54 | 0.32 – 7.42 | 0.590 |
| Context C1 * Sure loss | 1.49 | 1.19 – 1.88 | **0.001** |
| Context C2 * Sure loss | 0.79 | 0.63 – 0.99 | **0.040** |
| Amount * Sure loss | 0.60 | 0.48 – 0.76 | **<0.001** |
| Context C1 * Amount * Sure loss | 0.87 | 0.63 – 1.18 | 0.364 |
| Context C2 * Amount * Sure loss | 0.96 | 0.71 – 1.29 | 0.781 |
| N _id_ | 330 | | |
| Observations | 3960 | | |

The results show that there was a main effect of amount (54.19% and 55.61% sure loss choices for the low and high amount, respectively) and a main effect of Sure loss (58.79%, 58.18%, 56.21% 51.82%, 52.88%, 51.52% sure loss choices when the sure loss equaled, -0.5%, -1%, -3%, -5%, -10%, and -15%, respectively). The effect of amount and sure loss are similar to Experiment 2, although we see higher preferences for the sure losses. There was also an interaction between context and sure loss. We first looked at the effect of the sure loss in the NIR context, which was significant, *b* = .44, *SE* = 0.10, 95% CI [0.28, 0.64], *z* = 4.94, *p* < .001, *OR =* 1.56: as the sure loss increased, participants were more likely to choose the risky option. There was, however, no effect of the sure loss in the Base and Covid-19 contexts (both *p*s > .43). Nevertheless, it is worth noting that in these two contexts, the allocations to the sure loss option (independent of the sure loss size) never dipped below 50% hinting at indifference and risk aversion at the expense of the mixed gamble (see Figure 4).



***Figure 4.* Percentage of choices for the Risky and the Sure loss option in Experiment 3 as a function of the sure loss and the three contexts.** Numbers inside the bar charts represent the choice percentages.

Additionally, there was an interaction between amount and sure loss. Looking at the low amount ($100), there was an effect of sure loss *b* = .59, *SE* = 0.10, 95% CI [0.39, 0.80], *z* = 5.71, *p* = < .001, *OR =* 1.80 where, as the sure loss increases, participants were more likely to choose the risky option. For the high amount ($500), there was no effect of sure loss (*p* = .55).

Finally, we included the demographic variables into the main model and found a main effect of age, *b* = -.06, *SE* = 0.02, 95% CI [-0.09, -0.01], *z* = -2.28, *p* = .02, *OR =* 0.95 income, *b* = .38, *SE* = 0.17, 95% CI [0.04, 0.72], *z* = 2.17, *p* = .03, *OR =* 1.46 and financial experience *b* = 1.06, *SE* = 0.31, 95% CI [0.46, 1.66], *z* = 3.47, *p* = .001, *OR =* 2.84: that older people were more likely to choose the sure loss option while those with more income and financial experience (similar to experiment 2) were more likely to choose the risky option. The rest of the results remained the same as described above.

**General discussion**

In three experiments, using allocation (Experiments 1 and 2) and choice (Experiment 3) response formats, participants were asked to make hypothetical monetary decisions between a sure loss option and a risky mixed gamble that yielded a much more attractive expected outcome but also had the possibility of an even larger loss than the one in the sure option. Unsurprisingly, the more profitable the gamble, the more appealing it was (i.e., we observed more risk-taking in Experiment 1). Similarly, the larger the sure loss, the more participants were willing to take risks (Experiment 2). Critically, however, the current findings highlighted a high tolerance and even a preference for sure losses in certain cases. Allocations of money to and choosing the sure loss option never dipped below 36% in Experiments 1 and 2, and below 44% in Experiment 3. Due to our careful framing of the choices options, these results cannot be explained by a status-quo bias.

The results require a more careful unpacking with respect to (cumulative) prospect theory (CPT). The theory predicts a fourfold pattern of risk preference: risk aversion for gains and risk seeking for losses of high probability and risk seeking for gains and risk aversion for losses of low probability. The underweighting of high probabilities contributes to risk seeking in choices between probable and sure losses and CPT predicts that people prefer a larger uncertain loss to a smaller, but certain loss – a preference pattern dubbed the certainty effect. However, with moderate probabilities, the shape of the value function, due to loss aversion, predicts extreme aversion to mixed gambles.

In Experiments 1 (and 2), nine possible outcomes were presented for the gamble, each associated with a probability of occurrence. In Experiment 1, the mixed gamble contained extreme loss and gain outcomes that were associated with low probabilities (i.e., 2%). This is relevant because CPT predicts that people overweight rare events. Large, unlikely losses lead to loss aversion, so CPT predicts that people ought to prefer sure losses in these cases. The mixed gambles we used also had large, unlikely gains, but losses are given more weight compared to gains (i.e., loss aversion).

We can also formalize this numerically. Using the set of parameters as defined by CPT, one can calculate the value of each prospect as predicted by the theory. We used the parameters as reported by Tversky and Kahneman (1992) and Table S1 in the supplementary material reports the CPT values for each prospect in all three experiments. Consistent with the argumentation presented above, in Experiment 1, CPT predicts (i.e., a higher value is assigned) that people should prefer the sure loss in all options save for when the mixed gambles had an expected return of 5% and 10%. In that sense, the results of Experiment 1 are consistent with CPT. We saw that when the expected return of the mixed gamble was 3%, people started to prefer to allocate money to the mixed gamble. Overall, people avoided mixed gambles with high expected returns.

In Experiment 1 the sure loss was small (at -0.5%), choice options varied considerably with differing loss/gain probabilities, and there were differing risk premiums. In Experiment 2, risk premiums were held constant at 11% and CPT assigns higher value to the mixed gambles in all choice options, predicting that people should *not* prefer the sure loss in any of the choice options. However, for smaller sure losses of -0.5% and -1%, we observed slight preference and indifference indicating that people may be more comfortable with smaller sure losses than a gamble with a high expected return. However, for higher sure losses, preference switched to the risky mixed gamble as predicted by CPT.

A possible limitation of these two experiments could be that, for the mixed gamble, we only provided participants with an illustration of the final outcomes rather than how much they would lose or gain, respectively. As a result, people could have understood the mixed gamble as a series of gains. While this was rectified in Experiment 3 (where people were told exactly how much they would lose/gain) it is unlikely. If participants considered the outcomes in the mixed gambles as potential gains, CPT predicts that people should have uniformly preferred the mixed gambles. We see that this was not the case.

Finally, in Experiment 3, CPT predicts that people should prefer the sure loss when it is -0.5%, -1%, and -3%, but should switch their preference to the gamble when the sure loss is -5%, -10%, and -15%. Thus, as the sure losses increase, relatively more weight is given to them, predicting that mixed gambles ought to be preferred. This is even though (remember risk premiums are held constant) as the sure losses increase, possible losses associated with the mixed gamble also increase. This pattern was observed when we gave participants the NIR highlighting instructions. Perhaps this financial context cued in people to a different mindset. Our results in the other instruction types, however, do not show this and we saw that people were indifferent or preferred the sure loss for all the options offered, which suggests a general aversion to the mixed gambles (even though they had a positive expected return). Perhaps the NIR context highlighted a return-oriented perspective amplified by the financial nature of these instructions, explaining why we observed increased risk taking with higher sure losses (Breuer et al., 2020).

The question presents itself then, what could explain the switch towards preferring mixed gambles at some point if the risk premiums are held constant? In Experiment 2 (and 3 in the NIR instructions), we saw that people become more risk-seeking when the sure loss was -3% (-10% in Ex. 3) and in general as the sure loss increased (became more negative). A priori, three possible outcomes could have been observed: i) no effect, because the risk premium was held constant, ii) decreased risk-taking, because as the sure losses increase larger expected losses are associated with the risky option, or iii) increased risk-taking. With risk premiums held constant, one possible explanation for this preference switch could be related to the concept of diminishing sensitivity (a concept, along with loss aversion, used to explain the shape of the value function in prospect theory). Diminishing sensitivity posits that the impact of a change diminishes with its distance from the reference point (Erev et al., 2008; Tversky & Kahneman, 1992). This could explain why the same risk premium (11%) could be more meaningful as the sure loss becomes more negative (Ganzach & Wohl, 2018). For example, in the case of -3% sure loss with a CPT value of -44.88 (for a $1,000 investment), an 8% expected return with a CPT value of -10.44 could be more meaningful than in the case -1% sure loss with a CPT value of –17.07 and a 10% expected return with a CPT value of 2.46 (CPT values as shown in Table S1). Comparing the CPT values, going from -3% to 8% results in a larger change of 34.44 than going from -1% to 10% which results in a smaller change of 19.53^[[5]](#footnote-5)^.

The choice setup we used in our experiments is, to the best of our knowledge, somewhat unique in the current literature. While some research has presented non-zero mixed gambles to a sure loss, these mixed gambles did not have positive expected returns (Erev et al., 2010). Another study looked at non-zero mixed gambles and allowed participants to have state negative certainty equivalents (making it similar to our setup), but the study was more interested in comparing prospect theory parameters across time and didn’t report choice data (Zeisberger et al., 2012). Nevertheless, perhaps similar setups are present in other work and we cannot claim with certainty that these setups do not appear in the literature elsewhere.

There was a main effect of amount in Experiments 2 and 3 (higher allocations to the sure loss for the higher amount). In Experiment 3, for the low amount ($100) we found that as the sure loss increased, participants were more likely to choose the risky option, However, we found no such effect for the higher amount ($500) where participants generally preferred the sure loss (> 50% of choices) independent of the size of the sure loss (see Figure 4a in the OSF materials). This may again hint at the role of safety in these choices as participants may have been oriented to saving as much as possible as they could also stand to lose a lot more (relatively) when making choices with higher amounts.

In Experiment 1, we observed much larger allocations to the sure loss option in the decreasing than in the increasing order. Thus, in a world of decreasing opportunities, people seem to have gravitated towards the sure option, even though it entails a sure loss. The findings are somewhat in line with approaches that highlight decision from sampling where decisions are evaluated against a sample of other attributes using very simple cognitive processes such as ordinal comparison and frequency accumulation (Stewart et al., 2006). Thus, a return of 0.5 % (although positive) is seen as unattractive compared to a 10% return seen previously (see also Ert & Erev, 2013). However, we hamper any further speculation on this as we did not observe such effects in Experiment 2 and 3. This might be because in these experiments the changes were associated with the sure loss and not the risk premium, which was kept constant.

**Conclusion**

It thus seems that people were willing to pay for their patience (as in one year they would have less) as opposed to taking their chances and contending with a higher expected return down the line. In an understudied choice setup, we observed that people are quite tolerant of sure losses when the alternative is a risky prospect offering a much better expected return but also the possibility of an even larger loss. We also observed that this may depend on the choice context where in some cases, people preferred sure loses as high as -15%. The findings point to a lack of effectiveness of NIR policies in individual consumers. These policies, currently implemented by central banks and private banking organizations, aim to boost spending by penalizing saving (Agarwal & Kimball, 2019; Rogoff, 2017), but we see that people may be willing to tolerate these losses. This suggests that people may be impulsive in contexts that stress opportunities (i.e., a $100 in two days or $110 in a week), whereas they are willing to pay a cost for their patience in decision contexts where sure losses are made psychologically salient.

**Open practices statement**

Data and materials for all experiments are available at: <https://osf.io/3zfwu/?view_only=a85bbbdbe6ca42e6b8ae29428523afa5>

**References**

Abdellaoui, M., Bleichrodt, H., & Paraschiv, C. (2007). Loss Aversion Under Prospect Theory: A Parameter-Free Measurement. *Management Science*, *53*(10), 1659–1674. https://doi.org/10.1287/mnsc.1070.0711

Agarwal, R., & Kimball, M. (2019). Enabling Deep Negative Rates to Fight Recessions: A Guide. *IMF*, *Working paper No. 19/84*. https://www.imf.org/en/Publications/WP/Issues/2019/04/29/Enabling-Deep-Negative-Rates-A-Guide-46598

Baltussen, G., Post, T., & van Vliet, P. (2006). Violations of Cumulative Prospect Theory in Mixed Gambles with Moderate Probabilities. *Management Science*, *52*(8), 1288–1290. https://doi.org/10.1287/mnsc.1050.0544

Bartels, D. M., & Rips, L. J. (2010). Psychological connectedness and intertemporal choice. *Journal of Experimental Psychology: General*, *139*(1), 49–69. https://doi.org/10.1037/a0018062

Breuer, W., Soypak, C. K., & Steininger, B. I. (2020). *Conventional or Reverse Magnitude Effect for Negative Outcomes: A Matter of Framing* (SSRN Scholarly Paper ID 2176784). Social Science Research Network. https://doi.org/10.2139/ssrn.2176784

Erev, I., Ert, E., Roth, A. E., Haruvy, E., Herzog, S. M., Hau, R., Hertwig, R., Stewart, T., West, R., & Lebiere, C. (2010). A choice prediction competition: Choices from experience and from description. *Journal of Behavioral Decision Making*, *23*(1), 15–47. https://doi.org/10.1002/bdm.683

Erev, I., Ert, E., & Yechiam, E. (2008). Loss aversion, diminishing sensitivity, and the effect of experience on repeated decisions. *Journal of Behavioral Decision Making*, *21*(5), 575–597. https://doi.org/10.1002/bdm.602

Ert, E., & Erev, I. (2008). The rejection of attractive gambles, loss aversion, and the lemon avoidance heuristic. *Journal of Economic Psychology*, *29*(5), 715–723. https://doi.org/10.1016/j.joep.2007.06.003

Ert, E., & Erev, I. (2013). On the Descriptive Value of Loss Aversion in Decisions under Risk: Six Clarifications. *Judgment and Decision Making*, *8*(3), 213–235.

Gal, D. (2006). *A psychological law of inertia and the illusion of loss aversion*. *1*(1), 23–32.

Ganzach, Y., & Wohl, A. (2018). A behavioral theory of the effect of the risk-free rate on the demand for risky assets. *Journal of Behavioral and Experimental Economics*, *76*, 23–27. https://doi.org/10.1016/j.socec.2018.06.006

Harinck, F., Van Dijk, E., Van Beest, I., & Mersmann, P. (2007). When Gains Loom Larger Than Losses: Reversed Loss Aversion for Small Amounts of Money. *Psychological Science*, *18*(12), 1099–1105. https://doi.org/10.1111/j.1467-9280.2007.02031.x

Kahneman, D., & Tversky, A. (1979). Prospect Theory: An Analysis of Decision under Risk. *Econometrica*, *47*(2), 263–292. https://doi.org/10.2307/1914185

Lakens, D. (2013). Calculating and reporting effect sizes to facilitate cumulative science: A practical primer for t-tests and ANOVAs. *Frontiers in Psychology*, *4*(NOV), 863. https://doi.org/10.3389/fpsyg.2013.00863

Leclerc, F., Schmitt, B. H., & Dubé, L. (1995). Waiting Time and Decision Making: Is Time like Money? *Journal of Consumer Research*, *22*(1), 110–119. https://doi.org/10.1086/209439

Mischel, W. (2014). *The Marshmallow Test: Understanding Self-control and How To Master It*. Random House.

Payne, J. W. (2005). It is Whether You Win or Lose: The Importance of the Overall Probabilities of Winning or Losing in Risky Choice. *Journal of Risk and Uncertainty*, *30*(1), 5–19. https://doi.org/10.1007/s11166-005-5831-x

Redelmeier, D. A., & Tversky, A. (1992). On the Framing of Multiple Prospects. *Psychological Science*, *3*(3), 191–193. https://doi.org/10.1111/j.1467-9280.1992.tb00025.x

Rogoff, K. (2017). Dealing with Monetary Paralysis at the Zero Bound. *Journal of Economic Perspectives*, *31*(3), 47–66. https://doi.org/10.1257/jep.31.3.47

Ruggeri, K., Alí, S., Berge, M. L., Bertoldo, G., Bjørndal, L. D., Cortijos-Bernabeu, A., Davison, C., Demić, E., Esteban-Serna, C., Friedemann, M., Gibson, S. P., Jarke, H., Karakasheva, R., Khorrami, P. R., Kveder, J., Andersen, T. L., Lofthus, I. S., McGill, L., Nieto, A. E., … Folke, T. (2020). Replicating patterns of prospect theory for decision under risk. *Nature Human Behaviour*, *4*(6), 622–633. https://doi.org/10.1038/s41562-020-0886-x

Stewart, N., Chater, N., & Brown, G. D. A. (2006). Decision by sampling. *Cognitive Psychology*, *53*(1), 1–26. https://doi.org/10.1016/j.cogpsych.2005.10.003

Thaler, R. H., & Johnson, E. J. (1990). Gambling with the House Money and Trying to Break Even: The Effects of Prior Outcomes on Risky Choice. *Management Science*, *36*(6), 643–660. https://doi.org/10.1287/mnsc.36.6.643

Tversky, A., & Kahneman, D. (1992). Advances in prospect theory: Cumulative representation of uncertainty. *Journal of Risk and Uncertainty*, *5*(4), 297–323. https://doi.org/10.1007/BF00122574

Wu, G., & Markle, A. B. (2008). An Empirical Test of Gain-Loss Separability in Prospect Theory. *Management Science*, *54*(7), 1322–1335. https://doi.org/10.1287/mnsc.1070.0846

Zeisberger, S., Vrecko, D., & Langer, T. (2012). Measuring the time stability of Prospect Theory preferences. *Theory and Decision*, *72*(3), 359–386. https://doi.org/10.1007/s11238-010-9234-3

**Supplementary materials**

***Table S1.*** CPT values for the three experiments for each of the sure loss and mixed gamble prospects according to Cumulative Prospect Theory (CPT)^1^.

|  |  |  | CPT values | | | |
| --- | --- | --- | --- | --- | --- | --- |
| Ex1 |  |  | Investment is $1000 | | Investment is $40000 | |
|  | Sure loss | Expected return of gamble | Sure loss CPT | Gamble CPT | Sure loss CPT | Gamble CPT |
|  | -0.50% | -0.50% | -9.27 | -56.50 | -238.28 | -1451.55 |
|  | -0.50% | 0% | -9.27 | -52.18 | -238.28 | -1340.53 |
|  | -0.50% | 0.50% | -9.27 | -48.82 | -238.28 | -1254.40 |
|  | -0.50% | 1% | -9.27 | -45.56 | -238.28 | -1170.44 |
|  | -0.50% | 3% | -9.27 | -32.63 | -238.28 | -838.36 |
|  | -0.50% | 5% | -9.27 | -19.72 | -238.28 | -506.79 |
|  | -0.50% | 10% | -9.27 | 13.62 | -238.28 | 350.00 |
| Ex2 |  |  | Investment 1000 | | Investment 40000 | |
|  | Sure loss | Expected return of gamble | Sure loss CPT | Gamble CPT | Sure loss CPT | Gamble CPT |
|  | 0% | 11% | 0.00 | 9.06 | 0.00 | 232.71 |
|  | -0.50% | 10.50% | -9.27 | 5.74 | -238.28 | 147.51 |
|  | -1% | 10% | -17.07 | 2.46 | -438.53 | 63.25 |
|  | -3% | 8% | -44.88 | -10.44 | -1153.09 | -268.29 |
|  | -5% | 6% | -70.35 | -23.16 | -1807.54 | -594.99 |
| Ex3 |  |  | Investment is $100 | | Investment is $500 | |
|  | Sure loss | Expected return of gamble | Sure loss CPT | Gamble CPT | Sure loss CPT | Gamble CPT |
|  | -0.50% | 14.50% | -1.22 | -4.46 | -5.04 | -18.39 |
|  | -1% | 14% | -2.25 | -4.85 | -9.27 | -20.00 |
|  | -3% | 12% | -5.92 | -6.41 | -24.39 | -26.41 |
|  | -5% | 10% | -9.27 | -7.96 | -38.23 | -32.79 |
|  | -10% | 5% | -17.07 | -11.82 | -70.35 | -48.70 |
|  | -15% | 0% | -24.39 | -15.66 | -100.52 | -64.56 |

^1^Parameters used as in Tversky & Kahneman (1992): α = 0.88; β = 0.88; λ = 2.25; γ = 0.61;

δ = 0.69

1. Using [PANGEA](https://jakewestfall.shinyapps.io/pangea/), we determined that with this sample, we would have > 85% power to detect medium effect sized (*d* *=* .30) main between-subject effects and > 90% to detect medium effect sized (*d* *=* .30) main within subject effects. We would also have > 80% power to detect medium effect sized (*d* = .30) two-way and three-way interactions. Four-way interaction was not of interest. [↑](#footnote-ref-1)
2. We use the “dz” notation as suggested for standardized mean difference effect sizes for within-subject designs (Lakens, 2013). We stick with this notation throughout for consistency. [↑](#footnote-ref-2)
3. Adding order to our model, did not change the results and there were no main or interactive effects of order. [↑](#footnote-ref-3)
4. We used PANGEA to calculate sample size. To reasonably detect even a small effect size of *d =* .20 with > 80% power for the between subject condition we need around 110 participants per context condition (total N = 330). Power was more than >80% for all possible interactions. [↑](#footnote-ref-4)
5. A more formal possibility is that allocations to the sure loss option seemed to decrease as the *difference* between the sure and the *expected* loss associated with the risky option decreased*.* For example, the expected loss of the risky option, when the sure loss is -1%, amounts to $41 so the difference between the expected and the sure loss (i.e., $41 - $10) is $31. However, the expected loss of the risky option when the sure loss is -5% is $55.8, making the difference ($55.8-$50) of $5.8 much smaller. Perhaps due to the within-subject design, people were sensitive to this feature. To verify this, we compared allocation decisions of people who first saw the 0% sure loss (without seeing any other) and those that first saw the -5% sure loss in Experiment 2. Results still showed that people allocated less money in the sure loss option when it was -5% (34.7%), than when it was 0% (59.9%) (see supplementary material here: <https://osf.io/bw4ez/?view_only=ba75d8bb6c524b63bf34e0c273e1d107>). Unfortunately, we do not have data on which amount participants saw first in Experiment 3 so we cannot perform the same calculation for this experiment. [↑](#footnote-ref-5)
